# The Role of Ectonucleotidases CD39 and CD73 and Adenosine Signaling in Solid Organ Transplantation

**DOI:** 10.3389/fimmu.2014.00064

**Published:** 2014-02-18

**Authors:** Veena Roberts, John Stagg, Karen M. Dwyer

**Affiliations:** ^1^Immunology Research Centre, St. Vincent’s Hospital Melbourne and Department of Medicine, The University of Melbourne, Melbourne, VIC, Australia; ^2^Centre de Recherche du Centre Hospitalier de l’Université de Montréal, Faculté de Pharmacie et Institut du Cancer de Montréal, Montréal, QC, Canada

**Keywords:** adenosine, CD73, CD39, Treg, B cells

## Abstract

Extracellular adenosine is a potent immunomodulatory molecule that accumulates in states of inflammation. Nucleotides such as adenosine triphosphate and adenosine diphosphate are release from injured and necrotic cells and hydrolyzed to adenosine monophosphate and adenosine by the concerted action of the ectonucleotidases CD39 and CD73. Accumulating evidence suggest that purinergic signaling is involved in the inflammatory response that accompanies acute rejection and chronic allograft dysfunction. Modification of the purinergic pathway has been shown to alter graft survival in a number of solid organ transplant models and the response to ischemia–reperfusion injury (IRI). Furthermore, the purinergic pathway is intrinsically involved in B and T cell biology and function. Although T cells have traditionally been considered the orchestrators of acute allograft rejection, a role for B cells in chronic allograft loss is being increasingly appreciated. This review focuses on the role of the ectonucleotidases CD39 and CD73 and adenosine signaling in solid organ transplantation including the effects on IRI and T and B cell biology.

## Introduction

Solid organ transplantation is life sustaining and the preferred treatment for patients with end stage organ disease. Despite improvements in short term graft survival coincident with more potent immunosuppression, long term graft survival has not changed significantly. Although, the cause of chronic allograft dysfunction and failure has not been fully elucidated, evidence implicates recurrent episodes of acute allograft rejection in the pathogenesis. Life-long immunosuppression is a necessity in all but a few patients and a delicate balance exists between sufficient immunosuppressive to preserve graft integrity and unwanted side effects consequent to excess exposure. Immunological tolerance remains the panacea of transplantation medicine enabling long term allograft survival with the avoidance of immunosuppression toxicities.

A number of factors have been identified, which increase graft immunogenicity and the risk of rejection. Vast immunological disparity, evident in xenotransplantation, is a potent activator of the recipient’s immune system inciting an immediate, aggressive, and fulminant response termed hyperacute rejection (HAR). However, lesser antigen mismatches are also sufficient to trigger the alloimmune response, which may be classified histopathologically as cell mediated rejection (CMR) or antibody mediated rejection (AMR). The phenomenon of ischemia–reperfusion injury (IRI), an obligatory event in the transplantation process occurring at the time of organ procurement and engraftment, increases the risk of delayed graft function and immunogenicity of the graft particularly in the setting of extended cold preservation times. Strategies to curb the recipient’s immune response to such events without escalating immunosuppressive requirements remain the focus of intense research.

Current immunosuppressive regimens specifically target T cell activation as a means of preventing allograft rejection. The success of this approach is reflected in the exceptional 1 year graft survival of transplanted organs. However, despite the improvements in short term survival, long term graft survival remains static, and these agents are far less efficacious in combating chronic rejection. Recently, a role for B cells in chronic rejection has been appreciated; however, B cells may also be beneficial to the graft (1). B cells are able to modulate the T cell response by both enhancing the primary T cell response and promoting regulatory T cell (Treg) activity. These data have led researchers to conclude that functionally distinct B cell subsets exist classified as B effectors and B regulatory cells, respectively (2). The exact mechanisms by which B cells exert regulatory function are as yet unknown but putatively involve IL-10 which they produce (3).

Adenosine is an innate immunomodulatory molecule, the pericellular concentration of which rises dramatically in states of inflammation. Nucleotides such as adenosine triphosphate (ATP) and adenosine diphosphate (ADP) are extruded from injured and necrotic cells and hydrolyzed to adenosine monophosphate (AMP) by the ectonucleotidase NTPDase family of which CD39 (NTPDase1) is the prototype. AMP is then hydrolyzed by the 5′ ectonucleotidase CD73 to adenosine (4). Adenosine signals via four G protein coupled receptors namely A_1_, A_2A_, A_2B_, and A_3_. A_1_R and A_3_R are coupled to the G-inhibitory subunit which leads to a reduction in intracellular cAMP upon activation, whereas A_2A_R and A_2B_R are coupled to the G-stimulatory subunit resulting in an increase in intracellular cAMP. In addition, A_2B_R couples to Gq proteins, which stimulate phospholipase C activity and intracellular calcium mobilization (5). Originally described as a marker of B cell activation (6), CD39 expression has been demonstrated on resting B cells (2), T cells including Treg (7, 8), neutrophils (9), NK cells (10) monocytes, and macrophages (11). Similarly, CD73 was originally used as a surface marker to identify individual B cell subsets at specific stages of differentiation (12), but has since been demonstrated on resting B cells (2), T cells including Treg (13), neutrophils (9), NK cells (14) monocytes, and macrophages (11). CD39 and CD73 are co-expressed on resting B cells; however CD73 is down-regulated with activation (2). The ability to generate adenosine by these cells facilitates immunoglobulin diversification via class switch recombination, an essential process in mounting a humoral immune response (15) and which may impact graft survival. Within the T cell population CD39 and CD73 are co-expressed by Treg (8). Further, the adenosine receptor expression pattern has been detailed on both cellular subsets. The A_2A_R is expressed by T cells under basal conditions and is up regulated following activation (8). Recently, the importance of the A_2A_R on Treg function has been appreciated (16, 17). On B cells the A_1_, A_2A_, and A_3_R are all expressed although signaling via the A_3_R inhibits B cell proliferation (2).

There is mounting evidence that purinergic signaling is involved in the inflammatory response that accompanies rejection and in chronic allograft dysfunction. Modification of the purinergic pathway has altered graft survival experimentally in a number of solid organ transplant models and the response to IRI. Furthermore, the purinergic pathway is intrinsically involved in both B and T cell biology, cell subsets critical in maintaining allograft survival.

This review focuses on the role of ectonucleotidases (NTPDase1/CD39 and CD73) and adenosine signaling in solid organ transplantation including the effects on IRI and impact on lymphocyte biology.

## Cardiac Transplantation

Chronic cardiac allograft rejection manifests as coronary allograft vasculopathy (CAV), a rapidly progressive form of atherosclerosis that leads to reduced blood flow and ischemia and is a major cause of death in patients surviving more than 1 year after heart transplantation (18). Although numerous immune-mediated and metabolic risk factors have been implicated in the pathogenesis of CAV (19), to date no effective treatment is available to fully eliminate CAV and its related adverse outcomes. The main therapeutic strategy against CAV is the prevention and treatment of the factors known to trigger or accelerate the disease such as repeated episodes of acute allograft rejection and prolonged cold ischemia time associated with severe IRI.

### CD39, CD73, and A_2B_R mediates protection in cardiac ischemia–reperfusion injury

CD39 is a ubiquitously expressed integral immune and vascular ectonucleotidase and manipulation of this ectoenzyme is likely to impact on graft rejection in which inflammation and coagulation predominate. Indeed in the original description of mice deficient in CD39, CD39^−/−^ cardiac xenografts underwent rejection with more rapid vascular occlusion than did the matched wild-type (WT) murine hearts when grafted into rats (20). Thrombotic and inflammatory immunopathological changes were more evident in the mutant vasculature at earlier time-points compared with WT donor hearts. Replacement of CD39 in the form of adenovirus-mediated over-expression (21) or administration of apyrase (22), a soluble form of CD39, prolonged cardiac xenograft survival with reduced vascular thrombosis. Consistent with this the transgenic over-expression of CD39 improved cardiac xenograft survival with less platelet sequestration and preservation of cardiac architecture histologically (23).

NTPDase biochemical activity initially falls dramatically in the donor heart following transplantation but rebounds and CD39 mRNA expression is significantly increased in grafts that survive long term (24). Both human and murine CD39 mRNA expression has been documented to increase in a time dependent manner under hypoxic conditions and is dependent on the transcription factor Sp1 (25). Indeed, the phenomenon of ischemic preconditioning (IP), which involves multiple sublethal episodes of ischemia and reperfusion that augments adenosine concentrations and protects against further ischemia, robustly increases CD39 expression on endothelia and myocytes (26) (Table 1). Preconditioned WT hearts are significantly protected (26), whereas CD39 deficient hearts are more susceptible to myocardial infarction following coronary ischemia due to less ATP hydrolyzing ability (25). Conversely, mice treated with apyrase (26) or over-expressing CD39 (27) are protected against myocardial infarction following coronary artery occlusion through A_2B_R dependent mechanisms (27). These data implicate purinergic signaling pathways in the innate response to cardiac hypoxia and transplantation.

**Table 1:** **Expression of the ectonucleotidases and adenosine receptors on the organ parenchyma during ischemia–reperfusion injury**.

Organ parenchyma	Ectonucleotidase and adenosine receptors critical in IRI
Heart	CD39, CD73, A_2B_R
Trachea (lung)	A_2A_R
Liver	CD39, CD73
Kidney	CD39, CD73, A_2B_R

The A_2B_R is critical in mediating cardioprotection against hypoxic injury. Following 60 min of ischemia the area at risk was significantly less in mice treated with the A_2B_R agonist BAY 60-6583 (28) with the target gene being *Per2*, a circadian rhythm protein that enhances the glycolytic capacity of the ischemic heart through HIF-1α (29). The A_2B_R has been shown to increase post cardiac transplantation as early as 4 h following engraftment and parallels the changes in CD73 mRNA expression (14). Deficiency of CD73 in either the donor or the recipient reduced graft survival and accelerated the development of CAV in a murine model of cardiac transplantation and was associated with reduced A_2B_R expression (14). These data suggest that the expression of CD73 and A_2B_R are coordinated and dependent under conditions of cardiac hypoxia.

Cardiac transplantation involves a period of extended cold preservation when the donor heart is stored on ice during transportation to the recipient center. In a rat heterotopic heart transplant model administration of an adenosine bolus before cardioplegia and storage reduced myocardial injury and led to faster reanimation following reperfusion. Grafts treated with adenosine were less inflamed with fewer infiltrating cells (30).

### CD73 activity limits innate immunity following cardiac IRI

Bonner et al. (31) recently demonstrated that CD73 on circulating immune cells was critical in cardiac healing in a model of cardiac ischemia and reperfusion. Within 3 days of ischemia and reperfusion the myocardium was infiltrated with granulocytes and T cells, which highly express CD73 (32). The generation of adenosine was integral to limiting infarct size, inflammation, and the development of fibrosis (31). Although infarct size and cardiac ejection fraction were similar the day following ischemia, cardiac function continued to deteriorate in CD73 deficient mice whereas some recovery of function was observed WT mice. Lack of CD73 was associated with a sustained leukocytic myocardial infiltrate of a Th1 and M1 phenotype with enhanced expression of TNFα, IL-1β, IL-6, and IL-17 (31). Koeppen et al. (33) demonstrated CD73-generated adenosine mitigated inflammation and fibrosis preserving cardiac function through A_2B_R signaling specifically on polymorphonuclear cells which limited the release of TNFα.

These data suggest that adenosine is critical in the response of the heart to transplantation and augmenting peri-transplant adenosine levels reduces the impact of IRI and improves graft outcomes.

## Lung Transplantation

Chronic pulmonary allograft dysfunction manifests as bronchiolitis obliterans syndrome (BOS), which is characterized by progressive airflow obstruction and deterioration in function. BOS is a major complication following lung transplantation limiting long term survival. Ischemia–reperfusion injury, acute rejection episodes, and CMV infection are independent predictors of progressive BOS (34).

### A_2A_R activation attenuates lung ischemia–reperfusion injury

CD73 dependent generation of adenosine is essential in limiting inflammation occurring early following lung transplantation. Indeed greater inflammation was evident at 1 week in tracheal allografts transplanted into CD73 deficient recipient mice (35). The inflammatory infiltrate demonstrated increased CD3^+^ T cell infiltration and was accompanied by greater expression of Th1 cytokines IFNγ and IL-2 resulting in luminal narrowing. In the WT allografts, marked upregulation of A_2A_R mRNA expression was evident such that treatment of CD73 deficient recipients with an A_2A_R agonist rescued the allograft. In fact A_2A_R activation potently attenuates lung IRI if given before ischemia (36) or during reperfusion (37). Inflammation and pulmonary edema were maximally decreased and lung function optimized in a blood perfused rabbit-lung model subjected to ischemia followed by treatment with the specific A_2A_R agonist ATL313. When ischemia was combined with an A_2A_R inhibitor, this effect was abolished (36). Furthermore, cardiac dysfunction occurring concomitantly with pulmonary IRI is attenuated with A_2A_R activation (38). In mice A_2A_R activation reduces CD4^+^ T cell and neutrophil infiltration with marked reduction in inflammatory cytokines such as TNFα, IL-17, MCP-1, MIP-1, and RANTES (39) significantly improving lung function.

Human lung transplantation includes a period of cold preservation, which slows cellular metabolism limiting ischemic injury however; extended cold storage times increase the incidence of delayed graft function. A_2A_R activation decreases the inflammatory response and preserves pulmonary function following cold preservation and transplantation. Using a porcine transplant model, Reece et al. (37) demonstrated that treatment of the recipient pig with A_2A_R agonist beginning 10 min prior to and continuing for the first 3 h of reperfusion improved outcome. In this model, the porcine lung was subjected to 6 h of cold ischemia followed by 4 h of reperfusion. Animals treated with the intravenous infusion of A_2A_R agonist ATL-146e had improved oxygenation with preserved CO_2_ levels and acid–base balance. The measured pulmonary artery pressures and pulmonary vascular resistance were also lower in the A_2A_R agonist treated group. The overall lung injury score was significantly less primarily due to less pulmonary infiltration in the treatment group.

Human donor lungs may be perfused *ex vivo* with Steen Solution^®^ in order to optimize pulmonary function prior to engraftment. Based on the beneficial effect of A_2A_R activation in small animal warm ischemia–reperfusion models, Emaminia et al. (40) examined the effect of supplementing Steen Solution^®^ with A_2A_R agonist on pig explanted lungs which had been stored at 4°C for 5 h. Treated lungs demonstrated less edema, improved oxygenation index and mean airway pressure and lower levels of IFNγ, IL-1β, IL-6, and IL-18 suggesting A_2A_R agonist supplementation may further optimize lung function prior to transplantation. Indeed using a xenograft model, Westall et al. (41) demonstrated that genetically modified pig lungs lacking the αGal gene and expressing human complement regulatory proteins (CD55, CD59) and CD39 performed better *ex vivo* following perfusion with human blood. Lungs from genetically modified pigs demonstrated stable pulmonary vascular resistance, better oxygenation, and survived longer than WT lungs. Multiple potential factors may have contributed to the improved outcome including a putative effect of CD39 generated adenosine.

### A_2A_R activation attenuates acute allograft rejection in the lung

Recurrent episodes of acute allograft rejection promote the development of BOS. A_2A_R signaling is important in modifying the alloimmune response: A_2A_R activation reduces skin allograft rejection (42) and improves survival and functional engraftment of transplanted islets by inhibiting inflammatory islet damage in the peri-transplant period (43). Complete MHC mismatched tracheal allografts are rejected within 3 weeks manifesting with complete lumen obliteration. The kinetics of rejection was accelerated in A2ARKO mice with more inflammatory infiltrate comprising macrophages, neutrophils, and CD3^+^ T cells and collagen deposition. Conversely treatment of allograft recipients with A_2A_R agonist resulted in less leukocytic infiltrate and delay in luminal obliteration supporting an effect of A_2A_R activation in limiting ischemia–reperfusion injury (44). Intriguingly, a role for A_2B_R signaling has also been proposed in the development of BOS experimentally. A2BRKO mice recipient of a MHC mismatched tracheal allograft developed less severe BOS at 3 weeks (45). A greater number of FoxP3^+^ Tregs were present in the tracheal grafts in A2BRKO mice as early as day 3 post-op with concomitant reduction in neutrophils and CD4^+^ T cells, suggesting that A_2B_R activation may promote BOS via inhibiting Treg infiltration. The opposing effects of A_2A_R and A_2B_R signaling in tracheal transplantation may reflect the cellular expression of each adenosine receptor; the affinity for adenosine being approximately 50 times greater for the A_2A_R (ref) and that in addition to signaling via the G-stimulatory subunit and increasing intracellular cAMP, A_2B_R activation promotes calcium mobilization (ref).

Together, these data demonstrate evidence for A_2A_R activation in limiting acute pulmonary IRI and reducing alloimmune response with significant improvement in lung function following transplantation. On the other hand, A_2B_R signaling may promote allograft dysfunction indicating early inhibition may be therapeutically advantageous.

## Liver Transplantation

### CD39 and A_2B_R activity is protective in liver IRI

Liver transplantation remains the only therapeutic option for patients with end stage liver failure. The prevention of early graft dysfunction principally due to IRI is critical as there are limited supportive options available. As in other solid organs hepatic IP potently induces the transcription of CD39 via Sp1 (46) and CD73 (47), which promotes local adenosine generation protecting against subsequent prolonged ischemia. CD39 deficient (48) and CD73 deficient (47) mice are unable to be preconditioned and remain highly susceptible to the effects of prolonged ischemia with high mortality due to significant hepatic infarction.

Hypoxic preconditioning, like IP, confers protection against subsequent liver ischemia with marked attenuation of serum ALT, TNF-α, and IL-6 (49). This technique involves breathing 10% oxygen for 10 min prior to prolonged ischemia and results in a doubling of plasma adenosine concentrations. Employed in A2BRKO mice or inhibition of the A_2B_R negated the protective effect of hypoxic preconditioning providing the first evidence for the A_2B_R pathway in the prevention of warm hepatic IRI (49).

Deceased-donor and living-donor split-liver transplantation is a technique used to increase organ utility amongst potential recipients. The liver has a remarkable ability to regenerate after injury or resection and liver growth occurs at a rapid pace (50). CD39 plays a critical role in the regenerative capacity of the liver and is significantly attenuated in CD39 deficient mice (51). Stem cell mobilization directly correlates with restoration of liver volume and function and this is impaired in mice chimeric with CD39 deficient bone marrow (52).

### A_2A_R activation and CD39 activity reduces hepatic inflammation post IRI

Inflammation is an innate response to hepatic ischemia and reperfusion and T cells rapidly accumulate within the liver parenchyma. Administration of A_2A_R agonist during reperfusion significantly reduces liver injury following a period of ischemia, an effect lost in A2ARKO mice (53). Through a series of studies using chimeric mice, it was determined that it was the activation of the A_2A_R on circulating cells that conferred protection with less liver injury, reduced neutrophil infiltration, and induction of proinflammatory cytokine transcripts (54). These data were further refined by Lappas et al. (55), who demonstrated that the NKT cell subset of CD4^+^ T cells principally mediated early liver injury. Following 72 min of liver ischemia and 2 h of reperfusion CD1d-dependent activation of NKT cells lead to an increase in IFN-γ expression. Injury could be recapitulated in RAG-1 KO mice through the transfer of NKT cells and treatment of these cells prior to transfer with an A_2A_R agonist mitigated injury (55). However, activation of NKT cells *prior* to the ischemic insult actually confers protection. Cao et al. (56) activated NKT cells 1 h prior to ischemia and showed a reduction in neutrophilic infiltration and injury, which was both IL-13 and A_2A_R dependent.

Although these data implicate a pathogenic role for NKT cells in warm hepatic IRI this is not the case with cold IRI. Using a murine liver transplant model where the recipient mouse had undergone a total hepatectomy and was transplanted with either a donor liver from a WT or CD39 over-expressing mouse that had been stored at 4°C for 18 h, Pommey et al. showed significant protection in donor livers from CD39 over-expressing mice (57). In a series of chimeric studies and through detailed immunophenotyping of CD39 over-expressing mice the protective effect was attributed to a decrease in resident hepatic CD4^+^ T cells and the effect could be replicated through depletion of CD4^+^ T cells in WT mice. Notably, mice deficient in NKT cells remained susceptible to the effects of cold IRI. These data indicate significant differences in the pathogenetic mechanisms underlying warm and cold IRI associated with liver transplantation.

## Renal Transplantation

Renal transplantation remains the optimal form of renal replacement therapy for patients with end stage renal disease. Similar to other solid organ transplants long term survival is limited by the development of chronic allograft dysfunction which in the kidney manifests with interstitial fibrosis and tubular atrophy. Risk factors for late allograft loss include acute rejection that is increased in patients with delayed graft function and which in turn is impacted by IRI particularly with prolonged cold preservation times.

Evidence for the CD39-adenosinergic axis in renal IRI has been appreciated for some time and was recently reviewed (58). CD39 transcript expression is upregulated with IP (59) and CD39 deficient mice are highly susceptible to injury (60). Mice that over-express CD39 (61) or pre-treated with apyrase (59) are robustly protected from injury which is A_2B_R dependent (unpublished Karen M. Dwyer). As with other solid organs a period of cold ischemia often occurs in renal transplantation. Crikis et al. (61) examined the effect of CD39 over-expression in the donor kidney following a period of 5 h at 4°C. The donor kidney was transplanted into a bilaterally nephrectomized mouse recipient, which was culled 24 h later. Recipients receiving the CD39 over-expressing graft had better renal function as reflected by lower serum creatinine correlating with less tubular injury in the graft. These mice also remained healthy with preserved renal function up to 3 days post transplantation as opposed to mice receiving the WT kidney that did not survive to day 3 post renal transplantation (61).

Like CD39, CD73 is essential for preconditioning prior to renal ischemia (62). Intriguingly, Rajakumar et al. (63) described protection against injury in CD73 deficient mice. In this model, mice underwent 18 min of ischemia followed by 24 h of reperfusion, which induced mild injury in WT mice. Nevertheless, CD73KO mice were protected from injury and a similar result was obtained when WT mice were pre-treated with a CD73 inhibitor. These data implicate direct biological activity of AMP, which has recently been ascribed *in vitro* at the A_1_R (64).

With respect to adenosine signaling, A_2A_R activation mitigates inflammation inherent in this injury and parenchymal A_2B_R signaling improves post ischemic blood flow limiting injury (65, 66). CD4^+^ T cells are critical in mediating renal injury and pretreatment of mice with A_2A_R agonist significantly lessens this burden (67). As demonstrated in liver IRI, the CD4^+^ subset of NKT cells are activated rapidly after renal IRI, which is followed by the activation of other immune cells (68). Blocking dendritic cell mediated NKT cell activation via A_2A_R agonist treatment markedly reduced renal injury (69). Indeed, inhibition of adenosine receptor signaling is required for optimal dendritic cell, and subsequent T cell, activation (70). A series of elegant studies by Kinsey et al. (16, 17, 71) have emphasized the role of Tregs in inhibiting CD4^+^ T cell effector function through upregulation of PD-1 expression, which is augmented by A_2A_R activation.

Regulatory T cells traditionally have been defined by CD4 and CD25 co-expression and the transcription factor FoxP3. This subset have also been shown to express CD39 (7, 8, 72) and CD73 (8, 13) and the A_2A_R (8, 17). CD39 is essential for the full suppressive capabilities of these cells and mice deficient in CD39 are immunocompromised being more susceptible to T cell mediated diabetes (73) and experience more rapid skin graft rejection (8). In humans CD4, CD25, and CD39 expression identifies four T cell subsets, which can be followed longitudinally in the peripheral blood of renal transplant recipients (72).

## Summary

The process of solid organ transplantation involves ischemia–reperfusion injury that promotes immunogenicity of the donor organ leading to an increased risk of rejection and graft loss. Although T cells have been traditionally considered the orchestrators of these pathological processes, a role for B cells is being increasingly recognized. The recent identification and characterization of B cell subtypes; the recognition of dual but opposing effects on T cells; the putative involvement in allograft rejection make these cells a potentially attractive therapeutic target.

**Figure 1:**
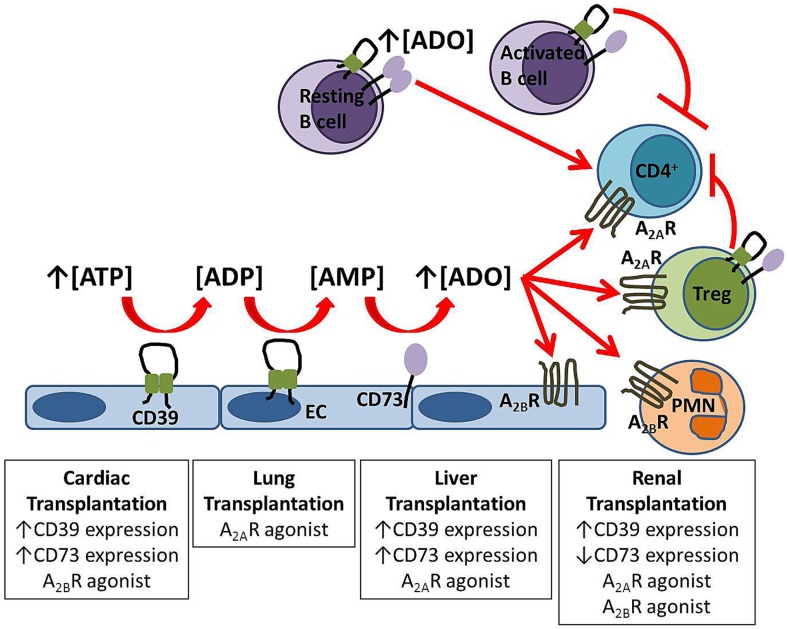
**Protective mechanisms in solid organ transplantation**. Extracellular adenosine is generated from the enzymatic hydrolysis of nucleotides by the ectoenzymes CD39 and CD73 expressed on endothelial cells (EC) and B cells. Adenosine signals via A_2A_R on circulating cells including regulatory T cells (Treg) and via A_2B_R expressed both on the vasculature and inflammatory cells. Experimental strategies which improve graft outcome for each solid organ transplant are listed in boxes.

The ectonucleotidases CD39 and CD73 are involved in adenosine generation and expressed by these important immune cell subsets. Adenosine signaling is integral to the function of both B and T cells, is protective in warm and cold ischemic injury and has immunomodulating properties, see Figure 1. There are current adenosine receptor agonists and antagonists being tested [reviewed in Ref. (74)] in non-transplant human trials but the ubiquitous nature of adenosine receptors make targeted therapy difficult. Potentially treatment of the donor organ prior to implantation with adenosine receptor agonists may reduce the impact of ischemia–reperfusion injury, subsequent acute rejection and graft failure.

## Conflict of Interest Statement

The authors declare that the research was conducted in the absence of any commercial or financial relationships that could be construed as a potential conflict of interest.
